# Pain Phenotype in Patients With Knee Osteoarthritis: Classification and Measurement Properties of painDETECT and Self‐Report Leeds Assessment of Neuropathic Symptoms and Signs Scale in a Cross‐Sectional Study

**DOI:** 10.1002/acr.22431

**Published:** 2015-03-25

**Authors:** Bryan J. Moreton, Victoria Tew, Roshan das Nair, Maggie Wheeler, David A. Walsh, Nadina B. Lincoln

**Affiliations:** ^1^ University of NottinghamNottinghamUK; ^2^ Universities of Lincoln and NottinghamNottinghamUK

## Abstract

**Objective:**

Multiple mechanisms are involved in pain associated with osteoarthritis (OA). The painDETECT and Self‐Report Leeds Assessment of Neuropathic Symptoms and Signs (S‐LANSS) questionnaires screen for neuropathic pain and may also identify individuals with musculoskeletal pain who exhibit abnormal central pain processing. The aim of this cross‐sectional study was to evaluate painDETECT and S‐LANSS for classification agreement and fit to the Rasch model, and to explore their relationship to pain severity and pain mechanisms in OA.

**Methods:**

A total of 192 patients with knee OA completed questionnaires covering different aspects of pain. Another group of 77 patients with knee OA completed questionnaires and underwent quantitative sensory testing for pressure–pain thresholds (PPTs). Agreement between painDETECT and S‐LANSS was evaluated using kappa coefficients and receiver operator characteristic (ROC) curves. Rasch analysis of both questionnaires was conducted. Relationships between screening questionnaires and measures of pain severity or PPTs were calculated using correlations.

**Results:**

PainDETECT and S‐LANSS shared a stronger correlation with each other than with measures of pain severity. ROC curves identified optimal cutoff scores for painDETECT and S‐LANSS to maximize agreement, but the kappa coefficient was low (κ = 0.33–0.46). Rasch analysis supported the measurement properties of painDETECT but not those of S‐LANSS. Higher painDETECT scores were associated with widespread reductions in PPTs.

**Conclusion:**

The data suggest that painDETECT assesses pain quality associated with augmented central pain processing in patients with OA. Although developed as a screening questionnaire, painDETECT may also function as a measure of characteristics that indicate augmented central pain processing. Agreement between painDETECT and S‐LANSS for pain classification was low, and it is currently unknown which tool may best predict treatment outcome.

## INTRODUCTION

Pain is a major symptom of patients with osteoarthritis (OA) and has a variety of characteristics suggesting differing underlying mechanisms 1. A range of approaches to pain management using analgesics, both those used in clinical practice and those in development, target discrete pain mechanisms. Heterogeneity between patients in the predominant mechanisms of OA pain may contribute to poor responses to treatment with specific agents. Valid tools are required to identify patients with OA who may respond to treatments targeting specific pain mechanisms.

Although OA is traditionally considered to be nociceptive, some patients describe aspects of their pain as burning or shooting. Such characteristics suggest mechanisms that are shared with neuropathic pain 2. The painDETECT questionnaire 3 and the Self‐Report Leeds Assessment of Neuropathic Symptoms and Signs (S‐LANSS) scale 4 were developed to help with the diagnosis of neuropathic pain. PainDETECT classifies subjects into groups based on a summative score for 9 items: neuropathic pain component is unlikely (score ≤12), result is ambiguous (score 13–18), and neuropathic pain component is likely (score ≥19). Most items use a 6‐point scale in which higher scores are suggestive of greater intensity. PainDETECT was originally developed for individuals with low back pain and showed good sensitivity (85%) and specificity (80%) when compared with a clinical diagnosis of pain of a predominantly nociceptive origin (e.g., visceral pain) or neuropathic origin (e.g., postherpetic neuralgia) 3.

S‐LANSS uses a binary response system requiring subjects to confirm whether or not they have experienced a symptom. It uses a summative score for 7 items to classify subjects into 2 groups: pain is not of a predominantly neuropathic origin (score <12) and pain is of a predominantly neuropathic origin (score ≥12). S‐LANSS exhibited good sensitivity (74%) and specificity (76%) when compared with clinical assessment of pain type across groups of individuals with primarily nociceptive conditions (e.g., headaches) or neuropathic conditions (e.g., nerve entrapment) 4. Hochman et al 5 compared a modified painDETECT questionnaire with the S‐LANSS scale in patients with knee OA and observed a strong positive correlation (ρ = 0.73, *P* < 0.0001). However, those investigators did not control for pain intensity and did not examine agreement between classifications.

Although painDETECT and S‐LANSS were developed to classify neuropathic pain, these instruments have also been used to measure neuropathic pain–like symptoms 6, 7. Associations of high painDETECT scores with a low (more sensitive) pressure–pain threshold (PPT) 8 suggest that these pain qualities are associated with augmented pain processing, even in persons without clinical evidence of neuropathy 9. PPTs are also reduced in patients with OA, both remote and distal from the affected joint, suggestive of augmented central pain processing, which is also known as central sensitization 10. Hochman et al 11 observed that patients with knee OA and modified painDETECT scores of >12 had an increased chance of displaying signs of central sensitization relative to patients with scores of <12. Central sensitization in knee OA may be attributable to chronically painful stimulation from the affected joint 5 and neuroimmune interactions in the central nervous system.

It is important to examine the measurement properties of the questionnaires, because both have been used as measurement scales. This can be achieved with Rasch analysis, which has several statistical advantages, including (given fit to the model) transformation of raw scores into interval‐level data, thereby facilitating, for example, valid analysis of changes in data following an intervention 12. An earlier version of LANSS was not shown to fit the Rasch model in patients with OA 13, but Rasch analyses of S‐LANSS and painDETECT have not been reported.

The aim of this study was to optimize and compare properties of painDETECT and S‐LANSS as classification and measurement questionnaires in patients with knee OA. Relationships between these questionnaires and measures of pain intensity were investigated to explore possible confounding by pain severity and their potential for use in phenotyping. Associations between painDETECT scores and PPTs were assessed to examine the extent to which this questionnaire measures augmented central pain processing.

Box 1 **Significance & Innovations**

- Although painDETECT and the Self‐Report Leeds Assessment of Neuropathic Symptoms and Sign may classify people with knee osteoarthritis (OA) according to a pain phenotype, potentially representing augmented central pain processing, the level of agreement between the classifications made by these questionnaires is low. Further work is required to establish the accuracy of these questionnaires as stratification tools in this condition.
- Rasch‐converted painDETECT scores may function as a measurement scale for the extent of augmented pain processing in people with knee OA.
- Questionnaires that classify pain mechanisms in people with OA could help target treatments to those who are most likely to benefit, and identify subgroups of patients suitable for recruitment to clinical trials.

## PATIENTS AND METHODS

Patients with radiographic evidence of knee OA and accompanying pain on most days for the past month were recruited to participate in a cross‐sectional questionnaire study between December 2010 and July 2011 (for review, see ref.^14^). Radiographs obtained from the patients were scored using the Kellgren/Lawrence system, and scores of ≥2 were used to define radiographic OA. Approximately half of the patients were identified from a previous study 15, and the remaining patients were recruited from clinics in Nottingham University Hospitals NHS Trust and Sherwood Forest Hospitals NHS Foundation Trust. Exclusion criteria were another inflammatory rheumatic condition (e.g., rheumatoid arthritis, psoriatic arthritis, gout, or fibromyalgia), knee joint surgery within the last 3 months, and an inability to understand English or otherwise complete the questionnaires.

Potential participants were sent an invitation to complete a questionnaire set covering different aspects of pain. Questionnaires were completed at home and returned by prepaid envelope. Data are reported from the McGill Pain Questionnaire (MPQ) pain rating index and present pain intensity scale 16, Intermittent and Constant Osteoarthritis Pain scale (ICOAP) 17, RAND 36‐Item Short Form Health Survey pain subscale 18, painDETECT, and S‐LANSS (including a 0–10 numerical rating scale for pain intensity). At the start of the study, the patients were informed that questions about their pain pertained to OA‐related knee pain 5. Patients with bilateral OA knee pain were permitted to choose which joint(s) to rate, to permit an evaluation of their overall pain experience. The patients were permitted to continue their usual medication regimen during the study. Approval was granted by Nottingham Research Ethics Committee One, and all participants provided informed consent.

### Pressure–pain thresholds

A separate sample of patients with clinically diagnosed OA and accompanying knee pain was recruited from primary and secondary care services through another questionnaire study, in order to measure PPTs; radiographs were not scored for these patients. As part of the questionnaire study, these patients were provided an information sheet that included a section on PPT testing and were invited to indicate on their consent form if they were interested in participating. Those interested were telephoned to confirm eligibility and to arrange a time for the test. The same exclusion criteria applied, but patients were also required to be able and willing to travel and undergo PPT testing. In addition to the larger questionnaire, the patients also completed painDETECT, a pain body map, and the ICOAP scale; all of these questionnaires were completed at home. On the day of testing (an average of 34 days after the questionnaires were administered), the patients rated their worst knee pain over the last week on a scale from 0 (no pain) to 10 (pain as severe as it could be) 4. Testing was performed between March 2012 and July 2013 at Nottingham City Hospital or Queen's Medical Centre.

PPTs were measured by 1 of 2 female researchers, using an electronic pressure algometer (SENSEBox; Somedic). The algometer had a 1‐cm–diameter probe, which was applied with increasing pressure at a rate of 50 kPa/second. When patients pressed a switch to indicate that the application was experienced as pain, the probe was immediately retracted, and stimulation ceased. Patients were familiarized with the testing procedure by application of the stimulus to their fingernail. PPTs were recorded at 3 sites in reference to their more painful knee 10: sternum, ipsilateral medial tibiofemoral joint line, and anterior tibia. The probe was placed on each site 3 times. To avoid temporal summation effects, there were 2‐minute rest intervals between testing at the 3 sites.

### Statistical analysis

Kappa coefficients 19 were used to examine agreement between painDETECT and S‐LANSS classifications 20. Receiver operating characteristic (ROC) curves were plotted to maximize agreement between questionnaires 21, 22. Kappa coefficients and ROC curves were calculated using SPSS version 21.0. In lieu of a gold standard for neuropathic pain 5, painDETECT and S‐LANSS were used as screening measures and as the standard for comparisons. The area under the curve was reported for each ROC curve, and the Youden Index and the point on the ROC curve closest to (0,1) 21 were used to identify optimal cutoffs. To explore relationships between painDETECT, S‐LANSS, and measures of pain severity, Spearman's correlation coefficients were calculated in SAS, version 9.1. Rasch‐converted scores were used in correlation analyses for both the questionnaire study and the PPT substudy, when available (for review, see ref. 14). Use of ICOAP subscale scores was supported by previous analyses 14. When appropriate, 95% confidence intervals (95% CIs) were calculated.

Rasch analysis 23 concerns assessment of fit between questionnaire data and predictions of the Rasch model. Several reviews 12, 24, 25 of Rasch analysis have detailed the process and defined terminology; therefore, only a brief overview is provided here. Analysis was conducted in RUMM2020 26 using the dichotomous and partial credit models 27. Summary fit residuals (mean ± SD) for items and persons and chi‐square testing for item–trait interactions were used to evaluate overall fit. Individual items and persons were also assessed for fit using standard criteria (i.e., 2.5> fit residuals >−2.5; chi‐square and analysis of variance [ANOVA] tests for items, with Bonferroni correction). Questionnaires were checked for disordered response thresholds, differential item functioning (DIF) for sex and age (age <64 years, age 64–71 years, and age >71 years), response dependency, and unidimensionality (for review, see ref. 14). The person separation index (PSI) was reported for each scale.

An arithmetic average was calculated for PPTs at each site and compared using Friedman's ANOVA by ranks, with pairwise comparisons. Associations between PPTs and Rasch‐transformed painDETECT scores used Spearman's correlation coefficients and partial correlations, controlling for constant and intermittent ICOAP scores. Demographics and pain scores were compared between the questionnaire study and the PPT substudy using Mann‐Whitney U and chi‐square tests. These tests were also conducted to compare patients included in the ROC curve analysis and those who were not, and to compare patients for whom there was agreement between painDETECT and S‐LANSS pain classifications and patients for whom there was no agreement. Patients with missing questionnaire data were included in the Rasch analyses of painDETECT and S‐LANSS and therefore Rasch‐based correlations with these questionnaires, because the Rasch model can handle missing data 28 (see Supplementary Table 1 and Table 2, available in the online version of this article at http://onlinelibrary.wiley.com/doi/10.1002/acr22431/abstract). These patients were not, however, included in the other analyses (e.g., ROC curves and correlations in the PPT substudy) or used with the other questionnaire scores.

Sample size calculations for the questionnaire study were based on Rasch analysis. Approximately 150 patients were required in order to have at least 95% confidence that item calibrations were within ±0.5 logits 29. A correlation of approximately −0.3 was expected between painDETECT and PPT sites, based on pilot study work. Eighty‐three patients would be needed to have 80% power for this effect size. Although fewer patients were entered into the substudy, the effect size was larger than anticipated.

## RESULTS

Of the 474 patients invited to take part in the questionnaire study, 192 were eligible and agreed to participate. Of the 171 patients who responded, 83 (49%) self‐reported pain localized to their knee(s) on the MPQ body map (27% unilateral and 21% bilateral), and 88 (51%) also reported pain in other areas. Additional characteristics of the patients are shown in Table 1. The painDETECT questionnaire was completed by 179 patients, but 29 had missing data. Twenty‐seven percent of the patients had a score of ≥19. S‐LANSS was completed by 180 participants, but 18 had missing data. A score of ≥12 was obtained by 30% of the patients. Complete data on both questionnaires were available for 135 patients (ROC subsample; see Table 1). There were no significant differences between demographic characteristics and the majority of pain scores for patients with and those without complete data on both screening questionnaires. However, available painDETECT scores were significantly higher for patients without complete data on S‐LANSS (*P* < 0.05).

**Table 1:** Demographic characteristics of the patients and descriptive statistics for the questionnaire study, the ROC subsample, and the PPT substudy*

	Questionnaire study (n = 192)	ROC subsample (n = 135)	PPT substudy (n = 77)
Patient characteristics			
Age, mean ± SD years	67 ± 10	67 ± 10	68 ± 9
Duration of pain, mean ± SD years	9 ± 9	10 ± 10	5 ± 5
Sex, % female	53	51	56
Questionnaire scores			
painDETECT (possible range −1–38)	13 (8–19)	13 (7–18)	13 (8–18)
S‐LANSS (possible range 0–24)	8 (2–13)	7 (2–13)	N/A
NRS (possible range 0–10)	7 (5–8)	7 (6–8)	7 (5–8)
MPQ PRI (possible range 0–78)	16 (11–24)	17 (11–25)	N/A
MPQ PPI (possible range 0–5)	2 (2–2)	2 (2–2)	N/A
ICOAP, constant (possible range 0–100)	45 (30–65)	45 (30–60)	53 (36–65)
ICOAP, intermittent (possible range 0–100)	50 (38–67)	50 (38–67)	54 (43–70)
RAND SF‐36 pain subscale (possible range 0–100)	45 (23–58)	45 (25–58)	N/A
PPT, kPa (possible range 0–1,600)			
Sternum	N/A	N/A	197 (118–300)
Medial tibiofemoral joint line^a^	N/A	N/A	235 (150–372)
Anterior tibia	N/A	N/A	159 (106–250)

Except where indicated otherwise, values are the median (interquartile range). Because of missing data, sample sizes used for calculating the descriptive statistics varied from 150 to 178 for the questionnaire study, from 115 to 135 for the receiver operating curve (ROC) subsample, and from 71 to 77 for the pressure–pain threshold (PPT) substudy. Missing data or unclear responses were recorded for 11–67 patients across the 3 samples. The duration of pain was based on patient self‐reports. The numerical rating scale (NRS) portion of the Self‐Report Leeds Assessment of Neuropathic Symptoms and Signs (S‐LANSS) questionnaire was completed on the day of test in the PPT substudy. MPQ = McGill Pain Questionnaire; PRI = pain rating index; PPI = present pain intensity; ICOAP = Intermittent and Constant Osteoarthritis Pain; RAND SF‐36 = RAND 36‐Item Short Form Health Survey.

a Values could not be obtained in 4 patients.

### Comparing classifications

Fair agreement 20 was observed for classification as probable neuropathic pain (κ = 0.35 [95% CI 0.16, 0.51], *P* < 0.001) using painDETECT and S‐LANSS cutoffs of ≥19 and ≥12, respectively. Although the questionnaires showed agreement for pain classifications in 100 patients (74%), they showed disagreement in 35 patients (26%). In 21 of these 35 patients, pain was classified by S‐LANSS as primarily neuropathic and by painDETECT as unlikely neuropathic or as an ambiguous result; for the remaining 14 participants, the opposite was observed. No significant differences were observed for demographic characteristics and the majority of pain scores between patients whose classifications were agreed upon and those whose classifications were not agreed upon. However, summary median painDETECT and S‐LANSS scores were significantly higher (both *P* < 0.001) in the group in which classifications were disagreed upon than in the group in which classifications were agreed upon, despite producing different pain classifications on an individual patient level in the former group.

**Figure 1:**
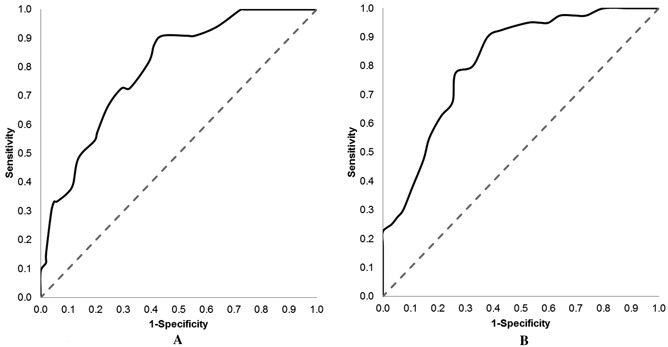
Self‐Report Leeds Assessment of Neuropathic Symptoms and Signs (S‐LANSS) (**A**) and painDETECT (**B**) receiver operating characteristic (ROC) curves for classification of neuropathic pain, as determined by the respective questionnaires. Each ROC curve indicates above‐chance performance (for S‐LANSS, area under the curve [AUC] 0.79, 95% confidence interval [95% CI] 0.71, 0.88; for painDETECT, AUC 0.82, 95% CI 0.74, 0.89), but no clear cutoff point.

ROC curves were constructed (Figure 1) to determine whether the published cutoffs could be altered to maximize classification agreement. Optimal cutoffs for painDETECT were >12 (90% sensitivity and 62% specificity) or >14 (78% sensitivity and 74% specificity), and those for S‐LANSS were >5 (91% sensitivity and 56% specificity) or >9 (73% sensitivity and 71% specificity), each using the Youden Index or the point on the ROC curve closest to (0, 1), respectively. These new cutoffs for painDETECT moderately improved classification agreement (κ = 0.42 [95% CI 0.29, 0.56], *P* < 0.001 and κ = 0.46 [95% CI 0.31, 0.61], *P* < 0.001, respectively) but the new cutoffs for S‐LANSS resulted in negligible change (κ = 0.33 [95% CI 0.21, 0.45], *P* < 0.001 and κ = 0.36 [95% CI 0.20, 0.52], *P* < 0.001, respectively).

### Rasch analysis of painDETECT

The item about the “course” of the patient's pain uses a negative scoring weight that cannot be entered into RUMM2020. Therefore, 1 was added to each of the scoring weights for this item. However, because this approach resulted in misfit (Table 2) and a high positive fit residual of 6.25, further analyses were conducted without this item.

**Table 2:** Fit statistics in the Rasch analyses of painDETECT and S‐LANSS*

Analysis	Item fit residual, mean ± SD	Person fit residual, mean ± SD	χ^2^(df) [*P*]	Person separation index	Percent of significant *t* **‐tests (95% CI)**
painDETECT					
All items	0.52 ± 2.33	−0.10 ± 0.99	102.67 (18) [0.05]	0.78	3.98 (0.80–7.20)
Minus item about course of pain	0.59 ± 1.10	−0.19 ± 1.05	19.82 (16) [0.23]	0.83	5.81 (2.60–9.10)
Subtest items referring to “burning” and “tingling”	0.71 ± 0.92	−0.16 ± 0.97	12.51 (14) [0.57]	0.82	4.68 (1.40–7.90)
S‐LANSS					
All items	0.19 ± 1.37	−0.03 ± 0.72	29.03 (14) [0.01]	0.68	0
Minus item 4	0.26 ± 0.86	−0.02 ± 0.73	22.45 (12) [0.03]	0.68	0
Minus item 6	0.35 ± 0.65	−0.02 ± 0.73	27.18 (10) [0.05]	0.59	a
Ideal values	0 ± 1	0 ± 1	[0.05]	>0.7	<5

a It was not possible to run the *t*‐test procedure in RUMM2020 for the last step of the Self‐Report Leeds Assessment of Neuropathic Symptoms and Signs (S‐LANSS) analysis due to the small set of items. Determination of the percent of significant *t*‐tests is used to examine unidimensionality of the scale. A principal components analysis of the residuals is conducted to identify the 2 most divergent subsets of items. These subsets are then used to generate separate person estimates that are compared using a series of *t*‐tests. In a unidimensional scale, no more than 5% of these tests should show significance at a level of 0.05. A binomial confidence interval (CI) is used for these analyses.

**Figure 2:**
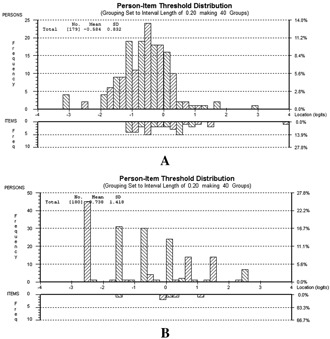
Person‐item threshold distributions for painDETECT (**A**) and Self‐Report Leeds Assessment of Neuropathic Symptoms and Signs (S‐LANSS) (**B**). The top part of each figure shows the distribution of persons along the Rasch‐estimated level of neuropathic pain (x‐axis). The lower part of each figure shows the distribution of items. Given good targeting, the 2 distributions should line up such that the range of neuropathic pain considered by the items matches the range of neuropathic pain expressed by the patients.

Two items exhibited marginally disordered thresholds (item 1, “burning sensation” in the painful areas; item 5, “cold or heat” is “occasionally painful”); however, because rescoring did not substantially affect fit, these items were left unaltered. Summary fit statistics indicated adequate fit and a unidimensional scale (Table 2). None of the remaining items misfit the model, but 5 patients had high negative fit residuals. Removal of these items did not particularly improve fit, and they were retained. For item 3 (“light touching” is painful), the expected values for women were higher than those for men, and for item 6 (“numbness” in the painful areas), the expected values for men were higher than those for women. Combining these items into a subtest resolved the DIF, indicating that the effects cancelled each other out; therefore, no remedial action was necessary. Items 1 and 2, which refer to similar sensations (“burning sensation” [e.g., stinging nettles] and “tingling or prickling”) exhibited response dependency. Grouping these items into a subtest improved fit (Table 3). Figure 2 shows that painDETECT was reasonably targeted. However, ∼20% of the patients had low trait levels, which were not measured.

**Table 3:** Final fit statistics for the individual items

Item	Location	SE	Fit residual	χ^2^	*P*	F	*P*
painDETECT							
1 & 2. “Burning sensation”/“tingling or prickling sensation”	−0.03	0.04	0.00	0.41	0.81	0.17	0.84
3. “Light touching” painful	0.34	0.08	−0.55	7.79	0.02	4.97	0.01
4. “Sudden pain attacks”	−0.63	0.06	0.58	0.79	0.67	0.67	0.52
5. “Cold or heat” painful	0.77	0.08	0.74	1.67	0.43	0.79	0.46
6. “Numbness” sensation	0.10	0.07	0.63	0.71	0.70	0.42	0.66
7. “Slight pressure” pain	−0.10	0.07	1.20	0.44	0.80	0.30	0.74
9. “Does your pain radiate”	−0.46	0.17	2.37	0.69	0.71	0.26	0.78
S‐LANSS^a^							
1. “Pins and needle” sensations	0.19	0.21	1.41	0.30	0.86	0.05	0.95
2. “Painful area change color”	1.13	0.23	0.85	3.83	0.15	1.27	0.28
3. “Skin abnormally sensitive to touch”	0.24	0.20	−0.65	7.07	0.03	4.95	0.01
5. “Burning pain”	−0.10	0.20	0.64	0.93	0.63	0.59	0.55
6. “Gently” rubbing feels different in “painful area”	−0.04	0.20	−0.74	7.19	0.03	5.16	0.01
7. “Gently” pressing feels different in “painful area”	−1.42	0.22	0.04	3.12	0.21	1.39	0.25

a S‐LANSS = Self‐Report Leeds Assessment of Neuropathic Symptoms and Signs.

### Rasch analysis of S‐LANSS

The summary fit residual for the items was high, and the chi‐square test value was significant (Table 2). The PSI was low and met only the minimum required value for group use when rounded to 1 decimal place 12. Item 4 (pain comes on “suddenly and in bursts”) had a high positive fit residual of 3.00. Removal of this item improved fit. Both the 7‐item S‐LANSS and the 6‐item S‐LANSS showed no misfitting persons and no evidence of response dependency, DIF, or multidimensionality. Item 6 (“gently” rubbing feels different in the “painful area”) was significant, as determined by ANOVA (F[2,116] = 5.16, *P* = 0.007) following removal of item 4 (Table 3). Because deletion of item 6 made both fit and the PSI worse, the item was retained. S‐LANSS was poorly targeted (Figure 2); there were gaps in measurement, meaning that the full range of the trait was not covered.

### Pain correlations

Rasch‐transformed painDETECT and S‐LANSS scores shared a stronger correlation with each other than with measures of pain severity (Table 4). The association between painDETECT and S‐LANSS remained strong after controlling for ICOAP scores for intermittent and constant pain (partial ρ = 0.58 [95% CI 0.46, 0.67], *P* < 0.0001). The correlations between the questionnaires were very similar when the standard scoring systems were used instead of the Rasch‐converted scores.

**Table 4:** Correlations between the questionnaires

	MPQ PRI	MPQ PPI	NRS	ICOAP, constant	ICOAP, intermittent	RAND SF‐36 pain subscale	painDETECT
MPQ PPI	0.35 (0.19, 0.49)^a^	–	–	–	–	–	–
NRS	0.35 (0.19, 0.48)^a^	0.40 (0.26, 0.53)^a^	–	–	–	–	–
ICOAP, constant	0.27 (0.12, 0.41)^b^	0.54 (0.41, 0.64)^a^	0.67 (0.58, 0.75)^a^	–	–	–	–
ICOAP, intermittent	0.31 (0.15, 0.45)^a^	0.42 (0.28, 0.54)^a^	0.62 (0.51, 0.71)^a^	0.74 (0.66, 0.80)^a^	–	–	–
RAND SF‐36 pain subscale	–0.39 (−0.52, −0.25)^a^	−0.46 (−0.57, −0.32)^a^	−0.61 (−0.69, −0.50)^a^	−0.70 (−0.77, −0.61)^a^	−0.66 (−0.73, −0.56)^a^	–	–
painDETECT	0.36 (0.21, 0.49)^a^	0.30 (0.15, 0.44)^b^	0.35 (0.21, 0.47)^a^	0.31 (0.16, 0.44)^a^	0.31 (0.17, 0.45)^a^	−0.30 (−0.43, −0.15)^a^	–
S‐LANSS	0.22 (0.06, 0.37)^b^	0.27 (0.11, 0.41)^b^	0.18 (0.03, 0.32)^c^	0.29 (0.14, 0.42)^a^	0.19 (0.04, 0.34)^b^	−0.23 (−0.37, −0.08)^b^	0.64 (0.54, 0.72)^a^

* Sample sizes varied from 139 to 177 for these calculations due to missing data. The correlation between painDETECT and Self‐Report Leeds Assessment of Neuropathic Symptoms and Signs (S‐LANSS) was similar when nontransformed scores were used (ρ = 0.62 [95% confidence interval (95% CI) 0.50, 0.71], *P* < 0.0001). Rasch‐converted scores were used for Intermittent and Constant Osteoarthritis Pain (ICOAP), RAND 36‐Item Short Form Health Survey (RAND SF‐36), painDETECT, and S‐LANSS. Values are Spearman's correlation coefficients (95% CIs [calculated using Fisher's Z transformation]). MPQ = McGill Pain Questionnaire; PRI = pain rating index; PPI = present pain intensity; NRS = numerical rating scale.

a *P* ≤ 0.0001.

b *P* ≤ 0.01.

c *P* ≤ 0.05.

### PPTs

Of the 114 patients contacted by telephone, 77 were included in the PPT substudy. Those who were not included either were not eligible or were unwilling to participate. Of the 76 patients who responded, 34 (45%) self‐reported pain only in their knee(s) (29% unilateral and 16% bilateral), and 42 (55%) also reported pain in other areas. Demographic characteristics are shown in Table 1. Patients in the PPT substudy did not differ significantly from those whose data were used for ROC curve analysis and Rasch analysis with respect to demographics or pain scores, except that patients in the PPT substudy reported a significantly shorter duration of pain (*P <* 0.05). The sternum and anterior tibia had significantly lower PPTs (more sensitive) compared with the medial joint line (*P* < 0.01). Higher painDETECT scores were associated with lower PPTs at each site, both in univariate analyses (sternum, ρ = −0.35 [95% CI −0.54, −0.13]; medial tibiofemoral joint line, ρ = −0.32 [95% CI −0.52, −0.08]; anterior tibia, ρ = −0.34 [95% CI −0.52, −0.11]; *P* ≤ 0.01 [n = 68−72]), and after controlling for ICOAP scores (sternum, ρ = −0.29 [95% CI −0.49, −0.06]; medial tibiofemoral joint line, ρ = −0.37 [95% CI −0.56, −0.14]; anterior tibia, ρ = −0.31 [95% CI −0.51, −0.08]; *P* ≤ 0.01 [n = 68–71]). Correlations between the PPT sites and the ICOAP (constant subscale ρ = −0.20 to −0.08, *P* > 0.05 [n = 72–76]; intermittent subscale ρ = −0.08 to 0.07, *P* > 0.05 [n = 73–76]) and NRS (ρ = −0.21 to −0.12, *P* > 0.05 [n = 73–77]) scores indicated that the associations were stronger between PPTs and painDETECT than between PPTs and measures of pain severity. Standard scores for painDETECT and ICOAP produced similar correlations.

## DISCUSSION

The characteristics of OA‐related pain are diverse, and the quality of this pain suggests mechanisms overlapping with neuropathic pain. In this study, we showed that painDETECT (with 1 item removed) may function as a measure of neuropathic pain–like symptoms in OA, but that S‐LANSS does not function well as a measurement scale. The pain characteristics measured by painDETECT were associated with widespread reductions in PPTs.

The mechanisms of OA pain remain incompletely understood but may include changes in the joint and changes in central pain processing 30. The current data confirm previous findings that symptoms characteristic of neuropathic pain are reported by some patients with OA 5. PainDETECT and S‐LANSS performed well as classification questionnaires in previous studies 3, 4 and categorized neuropathic pain in 27% and 30% of this sample, respectively. However, although each questionnaire classified neuropathic pain in similar proportions of patients, agreement between the questionnaires was only fair, with 74% of patients classified similarly by both questionnaires. Adjusting the cutoffs of either questionnaire had little impact on agreement. Tampin et al 31 reported similar results using LANSS and painDETECT in individuals with neck and upper limb pain (κ = 0.21). Further research is required to determine which tool provides the most accurate classification of pain mechanisms in OA.

PainDETECT, with removal of 1 item, displayed good fit to the Rasch model and was relatively well targeted to the sample, supporting its measurement properties. Although additional items that assess lower trait levels would be useful to fully capture the trait range, precision at this end of the scale is perhaps of reduced importance. Instead, focus should be on individuals with higher trait levels who may benefit from interventions addressing neuropathic pain–like symptoms.

S‐LANSS performed less well as a measurement scale. Misfitting items were observed, and the PSI was low, raising concerns about the reliability of the fit statistics and the ability of the scale to discern distinct strata within the sample 32. Furthermore, the scale was poorly targeted and would benefit from additional items. Similar results were observed in another study examining fit between the LANSS and the Rasch model 13, which suggests that both versions of this questionnaire may be best suited as screening tools.

Although some characteristics distinguish painDETECT and S‐LANSS, they shared a stronger association with each other than with measures of pain severity. The correlation between painDETECT and S‐LANSS remained strong when controlling for pain severity. Both questionnaires appear to address a coherent phenotype in patients with knee OA, despite the aforementioned differences in pain classifications. MPQ PRI scores exhibited similar associations with painDETECT, S‐LANSS, and measures of pain severity, possibly because the MPQ considers neuropathic‐like as well as nociceptive‐like characteristics 2, 33.

PainDETECT scores are increased in patients with fibromyalgia, a condition characterized by widespread pain and tenderness associated with abnormal central pain processing but without demonstrable neuropathology 8, 34. Widespread reductions in PPTs are common in patients with OA 10 or fibromyalgia 35 and reflect augmented central pain processing. This study showed that low PPTs across different body sites were associated with high painDETECT scores in patients with knee OA, even after adjusting for pain severity. PainDETECT scores have also been associated with pain thresholds in general population cohorts 9. High painDETECT scores may be a surrogate measure of augmented central pain processing 11 rather than necessarily indicating neuropathology in patients with OA. There was a time delay between questionnaire completion and PPT testing, which may have caused the strength of associations between painDETECT and PPTs to be underestimated. Further work could explore this by asking participants to complete the painDETECT questionnaire at the time of PPT testing.

Another limitation of the study is that non‐OA knee pain may have confounded the results. Individuals with central pain augmentation frequently report widespread pain, and 53% of the patients in this study reported pain at another site(s) in addition to the knee. Patients with known fibromyalgia (based on self report or case note review) were excluded, but the patients were not classified as having or not having fibromyalgia. Pain mechanisms in fibromyalgia may overlap with those in OA 36, and exclusion of patients with features of fibromyalgia could have biased the OA sample. Although the patients were asked to respond with reference to their knee pain 5, pain at other sites may have influenced responses to the generic pain questionnaires (e.g., the MPQ), which were not altered to be knee specific. It is not possible to infer causality from the associations in this cross‐sectional study, and further work is required to determine whether knee OA leads to neuropathology, whether alterations in central pain processing lead to pain characteristics shared with neuropathic pain, or whether a subgroup of patients are predisposed to experience knee OA with neuropathic symptomology. Further work is also required to determine whether the findings in patients with OA‐related knee pain are generalizable to other patient populations.

Neuropathic pain is classified as pain caused by a lesion or disease of the somatosensory nervous system 37. Neuropathy may contribute directly to OA knee pain through peripheral nerve damage within the joint 38, but there is currently no gold standard by which to identify neuropathy within articular nociceptive pathways. The screening questionnaires were originally validated by comparing patients in whom neuropathic pain was diagnosed by a physician and patients with arthritis and other nociceptive conditions 3, 4. Although symptoms may suggest neuropathy, it remains uncertain which questionnaire or which cutoff is most accurate in identifying neuropathic pain mechanisms in OA. In contrast, associations with PPTs at sites distant from the affected joints provide evidence that painDETECT scores reflect central pain processing in patients with knee OA. Central processing may augment pain severity and contribute to overlapping pain qualities associated with either nerve damage or joint damage.

In conclusion, ∼30% of the study sample had scores above the cutoff on painDETECT or S‐LANSS, potentially representing a phenotype within knee OA. This phenotype shares characteristics with neuropathic pain and is associated with augmented central pain processing, as indicated by widespread reductions in PPTs. The data suggest that painDETECT and S‐LANSS target a discrete phenotype in knee OA, reflecting pain quality as distinct from pain severity. In addition, Rasch analysis showed that painDETECT may be used as a measurement scale for these phenotypic characteristics. However, agreement between painDETECT and S‐LANSS for pain classification was only fair, and it is currently uncertain which tool is more accurate. Further work with a larger sample size is required to develop these questionnaires as stratification and outcome tools for testing treatments directed at central processing in patients with knee OA.

## AUTHOR CONTRIBUTIONS

All authors were involved in drafting the article or revising it critically for important intellectual content, and all authors approved the final version to be submitted for publication. Dr. Moreton had full access to all of the data in the study and takes responsibility for the integrity of the data and the accuracy of the data analysis.

**Study conception and design.** Moreton, das Nair, Walsh, Lincoln.

**Acquisition of data.** Moreton, Tew, Wheeler.

**Analysis and interpretation of data.** Moreton, das Nair, Walsh, Lincoln.

## Supporting information

Supplementary Table 1

Supplementary Table 2
