# Supplementary material for: Pain Phenotype in Patients With Knee Osteoarthritis: Classification and Measurement Properties of painDETECT and Self‐Report Leeds Assessment of Neuropathic Symptoms and Signs Scale in a Cross‐Sectional Study
Source: Arthritis Care Res (Hoboken). 2015 Mar 25;67(4):519–28. doi: 10.1002/acr.22431 (PMC4407932; doi:10.1002/acr.22431)
Supplement: Supplementary file 1 — Supplementary Table 1 [file ACR-67-519-s001.docx]

**SUPPLEMENTARY Table 1: PAINDETECT RASCH SCORES**

| Raw Score | ‘Rasch’ Score | Raw score (Continued) | ‘Rasch’ Score (Continued) |
| --- | --- | --- | --- |
| 0 | -3.14 | **19** | -0.01 |
| 1 | -2.40 | **20** | 0.07 |
| 2 | -1.94 | **21** | 0.16 |
| 3 | -1.65 | **22** | 0.25 |
| 4 | -1.44 | **23** | 0.35 |
| 5 | -1.27 | **24** | 0.44 |
| 6 | -1.14 | **25** | 0.55 |
| 7 | -1.01 | **26** | 0.66 |
| 8 | -0.91 | **27** | 0.77 |
| 9 | -0.81 | **28** | 0.90 |
| 10 | -0.72 | **29** | 1.04 |
| 11 | -0.64 | **30** | 1.21 |
| 12 | -0.56 | **31** | 1.40 |
| 13 | -0.48 | **32** | 1.63 |
| 14 | -0.40 | **33** | 1.94 |
| 15 | -0.33 | **34** | 2.40 |
| 16 | -0.25 | **35** | 3.24 |
| 17 | -0.17 | **36** | 4.80 |
| 18 | -0.09 |  |  |

*To convert painDETECT scores to interval-level data, do not include scores from the item about the ‘course’ of pain and if the participant’s pain radiates to other body regions add 1 instead of 2. Then calculate a total score ranging from 0 to 36 and look up the appropriate conversion score in the table.*
